# Germanium-based superatom clusters as excess electron compounds with significant static and dynamic NLO response; a DFT study†

**DOI:** 10.1039/d1ra08192f

**Published:** 2021-12-21

**Authors:** Atazaz Ahsin, Ahmed Bilal Shah, Khurshid Ayub

**Affiliations:** Department of Chemistry, COMSATS University Islamabad, Abbottabad Campus Abbottabad KPK 22060 Pakistan khurshid@cuiatd.edu.pk

## Abstract

Herein, the geometric, electronic, and nonlinear optical properties of excess electron zintl clusters Ge_5_AM_3_, Ge_9_AM_5_, and Ge_10_AM_3_ (AM = Li, Na, and K) are investigated. The clusters under consideration demonstrate considerable electronic stability as well as superalkali characteristics. The NBO charge is transferred from the alkali metal to the Ge-atoms. The FMO analysis shows fabulous conductive properties with a significant reduction in SOMO–LUMO gaps (0.79–4.04 eV) as compared with undoped systems. The designed clusters are completely transparent in the deep UV-region and show absorption in the visible and near-IR region. Being excess electron compounds these clusters exhibit remarkable hyperpolarizability response up to 8.99 × 10^−26^ esu, where a static second hyperpolarizability (*γ*_o_) value of up to 2.15 × 10^−30^ esu was recorded for Ge_9_Na_5_ superatom clusters. The excitation energy is the main controlling factor for hyperpolarizability as revealed from the two-level model study. The electro-optical Pockel's effect and the second harmonic generation phenomenon (SHG) are used to investigate dynamic nonlinear optical features. At a lower applied frequency (=532 nm), the dynamic hyperpolarizability and second hyperpolarizability values are significantly higher for the studied clusters. Furthermore, for the Ge_9_K_5_ cluster, the hyper Rayleigh scattering (HRS) increases to 5.03 × 10^−26^ esu.

## Abstract 2

Herein, the geometric, electronic, and nonlinear optical properties of excess electron zintl clusters Ge_5_AM_3_, Ge_9_AM_5_, and Ge_10_AM_3_ (AM = Li, Na, and K) are investigated.

## Graphical abstract

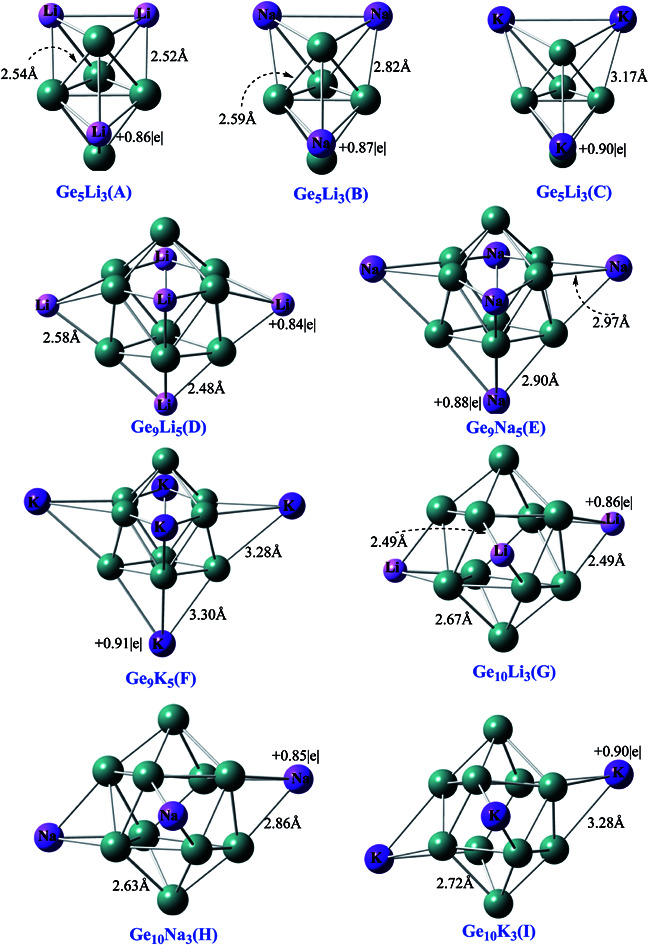

## 1 Introduction

The past several decades have witnessed increasing scientific and technology-driven interest in developing nonlinear optical (NLO) materials because of their tremendous importance in photonic applications.^1,2^ The developments in the field of nonlinear optics and laser-based technologies started after the discovery of the ruby laser by Maiman in 1960.^3^ Thus nonlinear optical materials have emerged rapidly during the last few decades, mainly due to their extensive applications in optoelectronic and photonic devices, second harmonic generation (SHG), endoscopy, and laser surgery^4–8^ To date, numerous approaches for designing nonlinear optical materials with high hyperpolarizability have been used, including diradical character,^9^ designing octupolar molecules,^10^ the push–pull effect in conjugated chromophores,^11^ multidecker sandwich complexes,^12^ and excess electron models.^13^ Among the studied nonlinear optical materials inorganic materials exhibit prime interest because of their physicochemical stability and thermal stability.^14^

The excess electron system is well-known for triggering second and third-order nonlinearity.^15,16^ In the family of excess electron compounds, electride,^17^ alkalides,^18^ and alkaline-earthides^19,20^ are well known.

Electrides are compounds in which electron trapping into the complexant acts as an anion.^21^ Similarly, the alkalides are compounds in which alkali metals possess a negative charge and become anion (Li^−^, Na^−^, K^−^).^22^ Alkaline-earthides are also a fabulous excess electron system that contains a negative charge on alkaline-earth metals (Be^−^, Mg^−^, Ca^−^).^23^ Excess electron compounds can be designed by doping any complexant with alkali metals,^24,25^ alkaline earth metals,^26^ transition metals^27,28^ and superalkali clusters.^29,30^

Superalkali clusters belong to the superatom clusters family and exhibit alkali-like characteristics with tunability in their electronic and geometric properties.^31^ The term ‘superalkalis’ was first time introduced by Gutsev and Boldyrev for Li_3_O, Li_2_F, NLi_4_*etc.* through DVMxα calculations. The superalkali clusters materials are of prime interest and show significant applications in numerous fields including, catalysis,^32^ reduction of CO_2_ and N_2_,^33^ hydrogen storage materials, and nonlinear optics.^34^ Thus, being excess electron compounds superalkali clusters can be adopted for making high-performance nonlinear optical materials. Furthermore, several studies have been proposed that reveal that superalkali being excess electron clusters can be doped with different molecules and nanocages to form excess electron compounds for triggering the NLO response. In this regard, superalkali-doped nanocages Li_3_O@Al_12_N_12_ were theoretically designed with electride characteristics for enhanced nonlinear optical response.^35^ Similarly, superalkali doped 2D graphdiyne M_2_X@GDY (where M = Li, Na, K, and X = F, Cl, Br) were studied, and it was observed that there is a significant decrease in the HOMO–LUMO gap with a notecable increase in hyperpolarizability response.^36^ Moreover, superalkali clusters were doped with different 2D materials and nanocages to get excess electron compounds *i.e.* alkalide, alkaline earthides. These excess electron compounds were reported as excellent nonlinear optical materials with significant hyperpolarizality.^24,37,38^ However, a very limited number of studies were reported that reveal the nonlinear optical response of pure superalkali clusters as efficient nonlinear optical materials.

A few number of pure superalkali clusters are investigated as excess electron compounds and show remarkable hyperpolarizability. In this regard, Misra *et al.*, investigated the electronic and nonlinear optical response of hyper lithiated superalkali clusters and reported these clusters as efficient NLO materials. The nonlinear optical response increases up to 1.2 × 10^4^ au.^39^ Subsequently, another class of superalkali clusters CNLi_*n*_ (*n* = 1–10) was investigated for nonlinear optical response and second-order NLO response was much pronounced.^40^ The literature reveals that only conventional types of superalkali clusters are explored for optical and nonlinear optical studies however several models can be efficiently utilized to be used as nonlinear optical responses. Literature also reveals, there are several superatom clusters (silicon-based) encapsulated by transition metals which were also studied for optical and magnetic excitation^41,42^

Zintl polyanions, discovered by Eduard zintl in 1930 belong to the group (14,15) and show excellent physicochemical stability.^43^ It is previously reported that zintl P_7_^3−^ anion as core material can be used to design organo-zintl superalkali clusters which contain superb electron properties.^44^ Similarly, zintl based superalkalis as a building block, when treated with superhalogens make novel supersalt compounds with significant electronic and nonlinear optical properties.^45^ Moreover, superalkali clusters other than alkali metals might possess better stability and a high nonlinear optical response. We become interested in zintl-based superatoms, particularly germinum-based superatoms (which belong to group 14 elements) for electronic and optical properties. Furthermore, the stoichiometry of these clusters obeys magic number nuclei, in which their valence shells are organised as 1S2, 1P6, 1D10, 2S2, 1F14 after losing one electron, achieving electronic shell closure (according to Jellium model). When designed with alkali metals, Ge semimetallic clusters may have improved optoelectronic and NLO features. Although the Ge_5_Li_3_, Ge_9_Li_5_, and Ge_10_Li_3_ were theoretically studied by Sun *et al.*^46^ Their investigations were limited to electronic properties whereas we adopted the alkali decorated zintl clusters Ge_5_AM_3,_ Ge_9_AM_5_ and Ge_10_AM_5_ (where AM = Li, Na, K) for exploring optoelectronic and nonlinear optical properties.

In these studied clusters, we are mainly concerned with the following issues. Do these clusters belong to superalkali with better thermal and electronic stability than conventional superalkalis do these clusters possess nonlinear optical responses for declaring them as efficient NLO materials. The NLO response was confirmed by hyperpolarizability calculation and second hyperpolarizability.

## 2 Computational details

Initially, all the studied alkali decorated zintl polyanions Ge_5_AM_3,_ Ge_9_AM_5,_ and Ge_10_AM_3_ (where AM = Li, Na, K) are considered and optimized at CAM-B3LYP/6-311+G(d,p) level of theory. The geometries of Ge_5_Li_3,_ Ge_9_Li_5,_ and Ge_10_Li_3_ were reported in the previous literature and we adopted the similar design for the rest of the alkali metals.^46^ All the calculations were performed with Gaussian 09 software.^47^ The CAM-B3LYP (Coulomb attenuating method) is a hybrid exchange–correlation functional that includes the hybrid properties of B3LYP functional and long-range corrected Coulomb-Attenuating Method (CAM).^48^ B3LYP is a hybrid part of the above method that contains Beckes 3-parameters for exchange functional and Lee–Yang–Parr-correlation functional. This hybrid density functional theory-based method comprises 0.19 HF plus 0.81 (B88) exchange interactions at short range and 0.65 HF plus 0.35 (B88) long-range interactions.^49^ The CAM-B3LYP is a well-known approach for linear and nonlinear optical characteristics of various clustered materials, and it has already been demonstrated to give appropriate geometries and comparable hyperpolarizability values with CCDST.^50–53^ Besides, the triple zeta split valence basis set 6-311+G(d,p) with diffuse and polarized function is adopted throughout the calculations.

To explore the electronic stabilities of these clusters, we calculated vertical ionization potential and electron affinity.

1 IE = *E*_X_^+^ − *E*_X_^0^

2 EA = *E*_X_^−^ − *E*_X_^0^

The chemical hardness is also calculated to understand their reactivity and soft nature and given by equation below ^54^

3 Chemical hardness (*η*) = VIP − VEA

To further explore the electronic properties, we performed frontier molecular orbital (FMOs) analysis which included SOMO, LUMO, and *E*_H–L_ gap. The FMOs analysis also provides evidence of the excess electron nature of studied superalkali clusters. Natural bonding orbitals (NBO) study is conducted to explore the nature and charge distribution as studied superatom clusters. The time-dependent density functional (TD-DFT) theory is adopted to calculate the excited state parameters and absorbance behavior of studied zintl superatom clusters. The time-dependent density functional (TD-DFT) is well know for obtaining excited states parameters and absorption spectra of molecules and clusters. The TD-DFT was chosen because of its performance and its correspondence to experimental results. For this purpose excited-state calculations with TD-CAM-B3LYP/6-311+G(d,p) are performed. Mathematically system under the constant field can be expressed as:4
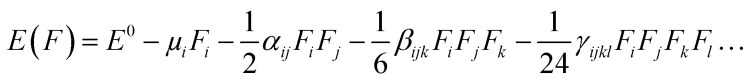
where *F* is an external applied electric field on the molecular system, *F*_*i*_ is its component of force along *i* direction; *E*^0^ is the total energy of the superalkali clusters without a static electric field, *μ*_*i*,_*α*_*ij*_, *β*_*ijk*_, and *γ*_*ijkl*_ are dipole moment, polarizability, hyperpolarizability, and second-order hyperpolarizability, respectively. Thus regarding nonlinear optical properties, the following parameters are estimated including the mean dipole moment (*μ*_o_), change in dipole moment (Δ*μ*), static polarizability (*α*) and static first hyperpolarizability (*β*).

5 *α*_o_ = 1/3(*α*_*xx*_ + *α*_*yy*_ + *α*_*zz*_)

6
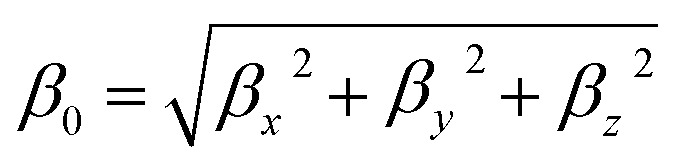
where *β*_*x*_ = *β*_*xxx*_ + *β*_*xyy*_ + *β*_*xzz*_, *β*_*y*_ = *β*_*yyy*_ + *β*_*yzz*_ + *β*_*yxx*_ and *β*_*z*_ = *β*_*zzz*_ + *β*_*zxx*_ + *β*_*zyy*_.

7 *μ*_o_ = (*μ*_*x*_^2^ + *μ*_*y*_^2^ + *μ*_*z*_^2^)^½^

Besides, the second static hyperpolarizability (*γ*_o_) and the projection of hyperpolarizability on the dipole moment vector (*β*_vec_) is also calculated for our studied superalkali clusters at the same level of theory. Vector part of hyperpolarizability (*β*_vec_) and second hyperpolarizability (*γ*) are expressed defined as

8 〈*γ*〉 = 1/5(*γ*_*xxxx*_ + *γ*_*yyyy*_ + *γ*_*zzzz*_ + *γ*_*xxyy*_ + *γ*_*xxzz*_ + *γ*_*yyxx*_ + *γ*_*yyzz*_ + *γ*_*zzxx*_)

9
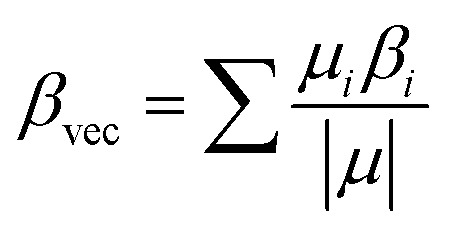


Mathematically *β*_HRS_ can be expressed as;10
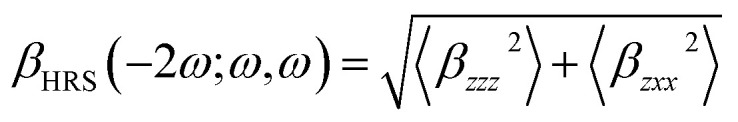
where 〈*β*_*zzz*_^2^〉 and 〈*β*_*zxx*_^2^〉 are average of orientational (*β*) tensor.

While the related depolarization ratio for these superalkali clusters (DR) ratio is also given by;DR = 〈*β*_*zzz*_^2^〉/〈*β*_*zxx*_^2^〉

The frequency-dependent NLO analysis was conducted at 532 and 1064 nm wavelength. Frequency-dependent hyperpolarizability involves the electro-optic Pockel's effect (EOPE) *β*(−*ω*;*ω*,0) and electric field induced second harmonic generation (ESHG) *β*(−2*ω*;*ω*,*ω*) respectively. While for second hyperpolarizability (*γ*), dc-Kerr *γ*^dc-Kerr^ (*ω*) = *γ*(−*ω*;*ω*,0,0) and second harmonic generation *γ*^ESHG^(*ω*) = *γ*(−2*ω*;*ω*, *ω*,0) were considered.

## 3 Results and discussion

### 3.1 Geometries and electronic properties

**Fig. 1:**
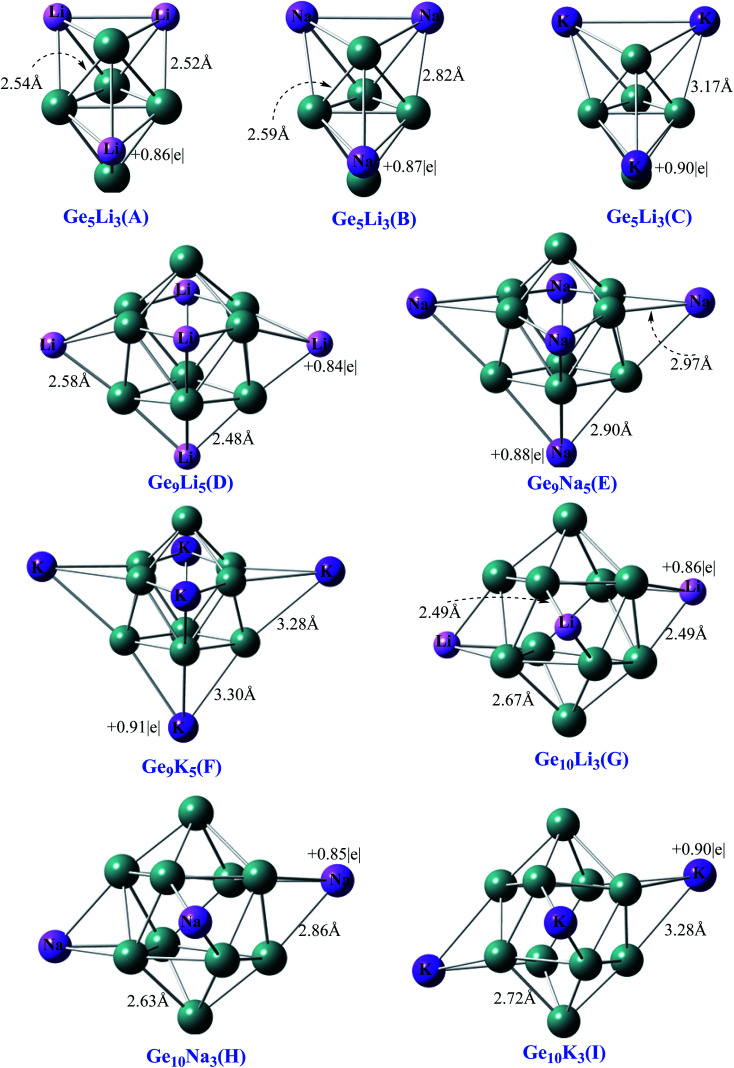
Optimized geometries with NBO charge of Ge_5_AM_3_, Ge_9_AM_5_ and Ge_10_AM_3_ clusters.

All the optimized geometries of Ge_5_AM_3_, Ge_9_AM_5,_ and Ge_10_AM_3_ (where AM = Li, Na, K) zintl superalkali clusters and their equilibrium bond length are depicted in Fig. 1. The geometric parameters of these clusters are identical to those reported in the earlier literature^46^ and are included in the ESI† (S1).

These clusters have no imaginary frequency (negative frequency) according to the frequency calculations, hence they are true minima on the potential energy surface (PES).

The computed ionization potential and electron affinity are used to analyze the electronic stability and superalkali nature of the investigated clusters. The difference in ground state energy between the cationic and neutral systems is described as the ionization potential (IP), meanwhile, the difference in ground state energy between the neutral and anionic systems is defined as the electron affinity.

The vertical ionization potential (VIP) values for Ge_9_AM_3_ are in the range of 5.49 to 3.91 eV, and it decreased dramatically as the alkali-metal size rose (Li to k). The VIP values for the Ge_9_AM_5_ series follow a similar pattern, reaching to 2.15 eV for F (Table 1). On the other hand, the VIP values for Ge_10_AM_3_ are slightly higher than for Ge_9_AM_5_ (Table 1). The lower VIPS values for the Ge_9_AM_5_ series can be attributed to the higher number of alkali metals (AM = 5), which is responsible for the cluster's electropositive nature. Additionally, their superalkali nature is demonstrated by their relatively low ionization potential values than Li atoms (5.4 eV).

**Table 1:** Vertical ionization potential (VIP, in eV), vertical electron affinity (VEA in eV), maximum chemical hardness (*η* in eV), average NBO charge upon germanium (*Q*_Ge_ in |*e*|), an average charge upon alkali metal (*Q*_AM_ in |*e*|), of Ge_5_AM_3_ Ge_9_AM_5_ and Ge_10_AM_3_ (where AM = Li, Na, K)

Superalkalis	VIP	VEA	*η*	*Q*_Ge_	*Q*_AM_
**Ge**_**5**_ **AM**_**3**_ **(where AM = Li, Na, K)**					
Ge_5_Li_3_ (A)	5.49	0.98	4.51	−0.69	0.86
Ge_5_Na_3_ (B)	4.69	0.64	4.05	−0.65	0.87
Ge_5_K_3_ (C)	3.91	0.29	3.62	−0.65	0.90

**Ge**_**9**_ **AM**_**5**_					
Ge_9_Li_5_ (D)	4.36	0.65	3.71	−0.61	0.85
Ge_9_Na_5_ (E)	2.81	0.07	2.74	−0.52	0.88
Ge_9_K_5_ (F)	2.15	0.02	2.13	−0.50	0.90

**Ge**_**10**_ **AM**_**3**_					
Ge_10_Li_3_ (G)	4.98	1.15	3.83	−0.38	0.86
Ge_10_Na_3_ (H)	4.34	0.67	3.67	−0.36	0.85
Ge_10_K_3_ (I)	3.50	0.11	3.39	−0.46	0.90

The calculated vertical electron affinity (VEA) values of these superalkali clusters range from 0.02 to 1.15 eV. In the designed series, Ge_9_AM_5_ has a smaller VEA value than those of Ge_3_AM_3_ and Ge_10_AM_3_. The electro-positive feature of the examined superalkalis is revealed by the tiny VEA values. Furthermore, the reduced VEA values indicate that these alkali-decorated zintl clusters are unable to completely grasp the valence (loosely bound) electron, which could result in interesting electrical properties. Table 1 shows that the examined clusters are polarizable and soft, based on the computed minimal values of chemical hardness. The calculated chemical hardness (*η*) values decrease with increased alkali metals size within these clusters. Among the series, Ge_9_AM_5_ clusters shows lower values of chemical hardness which may be attributed to the higher number of alkali smaller metals (soft) in these clusters.

### 3.2 Natural bonding orbital (NBO) analysis

The NBO analysis is a useful tool for interpreting intramolecular and intermolecular interactions and conjugative interactions in compounds and clusters.^55^ For Ge_5_AM_3_ clusters, the obtained charges on alkali metals (QAM) vary from 0.86 to 0.90|*e*| (positive magnitude). The C cluster has the highest computed positive charge of 0.90|*e*|, while A has the lowest value of 0.86|*e*| in the Ge_5_AM_3_ family. Similarly, the Ge_9_AM_5_ clusters, computed average charge (QAM) ranges from 0.85 to 0.90|*e*| (Table 1). For the Ge_10_AM_3_ superalkali clusters, similar NBO (positive magnitude) charges are observed. Hence, the computed positive NBO charges upon alkali metals shows the significant charge transfer (from alkali metals to Ge-atom) within the superalkali clusters. Additionally, the compted NBO charges upon germanium metals (*Q*_Ge_) for Ge_5_AM_3_ range from −0.13 to −0.69|*e*| (negative in magnitude). Therefore from the computed NBO charges (Table 1) one can observe the excellent separation of charges within the clusters. Likewise, the computed NBO charges (*Q*_Ge_) for the Ge_9_AM_3_ clusters are range from −0.09 to −0.61|*e*| where the highest value of −0.61|*e*| is obtained for Ge_9_Li_5_ while the lowest value (−0.50) is obtained for Ge_9_K_5_ superalkali. However, the reported NBO charges (*Q*_Ge_) for the third series Ge_10_AM_3_ are reduced (in negative magnitude) with corresponding increased cluster size. The overall NBO charges is an order of Ge_5_AM_3_ > Ge_9_AM_5_ > Ge_10_AM_3_.

### 3.3 Frontier molecular orbital (FMO) analysis and excess electron character

FMO analysis is adopted to validate the reactivity of the studied cluster system. Thus according to the frontier molecular orbital treatment of chemical reactivity, the rate, and site of reactivity of a molecule with a nucleophile is dominated by the interaction of the LUMO of the molecule in question with the HOMO of the nucleophile. The closer these orbitals are in energy, the more intensely they will interact, and the higher is the reactivity will be. Result in Table 2 show that the computed values for singly occupied molecular orbitals (SOMO), LUMO, and *E*_S–L_ gaps. For the Ge_5_AM_3_, the obtained values of singly occupied molecular orbitals (SOMO) vary from −4.99 to −3.51 eV and increase from A to C. For Ge_9_AM_5_, predicted SOMO energies are in the range of −3.97 to −1.80 eV, as well. The Ge_10_AM_3_ clusters, on the other hand, have lower SOMO energies than those of Ge_9_AM_5_ clusters, and these range from −4.46 to −3.14 eV. It can also be concluded that the SOMO energies of the examined superalkali clusters rise monotonically as the size of the alkali metals increases. Thus the observed trend of SOMO energies for studied superalkali clusters is Ge_9_AM_5_ < Ge_10_AM_3_ < Ge_5_AM_3_. Furthermore, the estimated LUMO energies for Ge_5_AM_3_ clusters vary with cluster size. However, the estimated energies of virtual orbitals for the Ge_9_AM_5_ series are increasing, while the Ge_10_AM_3_ clusters are increasing even high.

**Table 2:** Energies of SOMO and LUMOs (in eV), HOMO–LUMO gaps (*E*_H–L_ in eV), excitation energies (Δ*E* in eV), the wavelength of maximum absorbance (*λ*_max_ in nm), oscillator strength (*f*_o_ in au), ground-state dipole moment (*μ*_o_ in au), and excited-state dipole moment (Δ*μ* in au) of Ge_5_AM_3_, Ge_9_AM_5_ and Ge_10_AM_3_ superalkali clusters

Superalkalis	SOMO	LUMO	*E*_H–L_	Δ*E*	*λ*_max_	*f*_o_	*μ*	Δ*μ*
**Ge**_**5**_ **AM**_**3**_ **(where AM = Li, Na, K)**								
Ge_5_Li_3_ (A)	−4.99	−0.94	4.04	2.16	571	0.015	1.63	1.05
Ge_5_Na_3_ (B)	−4.24	−0.99	3.25	2.16	572	0.015	2.26	1.14
Ge_5_K_3_ (C)	−3.51	−0.73	2.77	2.24	553	0.041	3.12	2.34

**Ge**_**9**_ **AM**_**5**_								
Ge_9_Li_5_ (D)	−3.97	−0.07	3.89	2.25	548	0.007	0.27	0.62
Ge_9_Na_5_ (E)	−2.42	−1.07	1.34	1.77	688	0.076	1.69	2.49
Ge_9_K_5_ (F)	−1.80	−1.02	0.79	1.12	1101	0.219	2.21	1.86

**Ge**_**10**_ **AM**_**3**_								
Ge_10_Li_3_ (G)	−4.40	−0.87	3.73	2.31	534	0.007	1.45	0.62
Ge_10_Na_3_ (H)	−4.00	−0.83	3.16	2.47	500	0.008	1.55	0.65
Ge_10_K_3_ (I)	−3.14	−0.69	2.45	2.14	578	0.005	1.67	0.49

For Ge_5_AM_3_ clusters, there is a considerable reduction in the SOMO–LUMO gap, with values ranging from 4.06 to 2.77 eV. The HOMO–LUMO gaps for Ge_5_AM_3_ clusters are around 4.06 to 2.77 eV, indicating that these compounds behave like semiconductors. Similarly, the observed reduction in Ge_9_AM_5_*E*_H–L_ values from 3.89 to 0.79 eV could be attributable to their higher SOMO energies. Furthermore, the Ge_10_AM_3_ series' HOMO–LUMO gap values are slightly higher than the Ge_9_AM_3_ series, and range from 3.37 to 2.45 eV (Table 2). The significant reduction in HOMO–LUMO gaps reveals the soft nature (higher reactivity) and conductive properties of these clusters. Furthermore, the observed reduced SOMO–LUMO gap within clusters is attributed to the larger size of alkali metals (ease in the transition of electron from occupied to virtual orbitals).

**Fig. 2:**
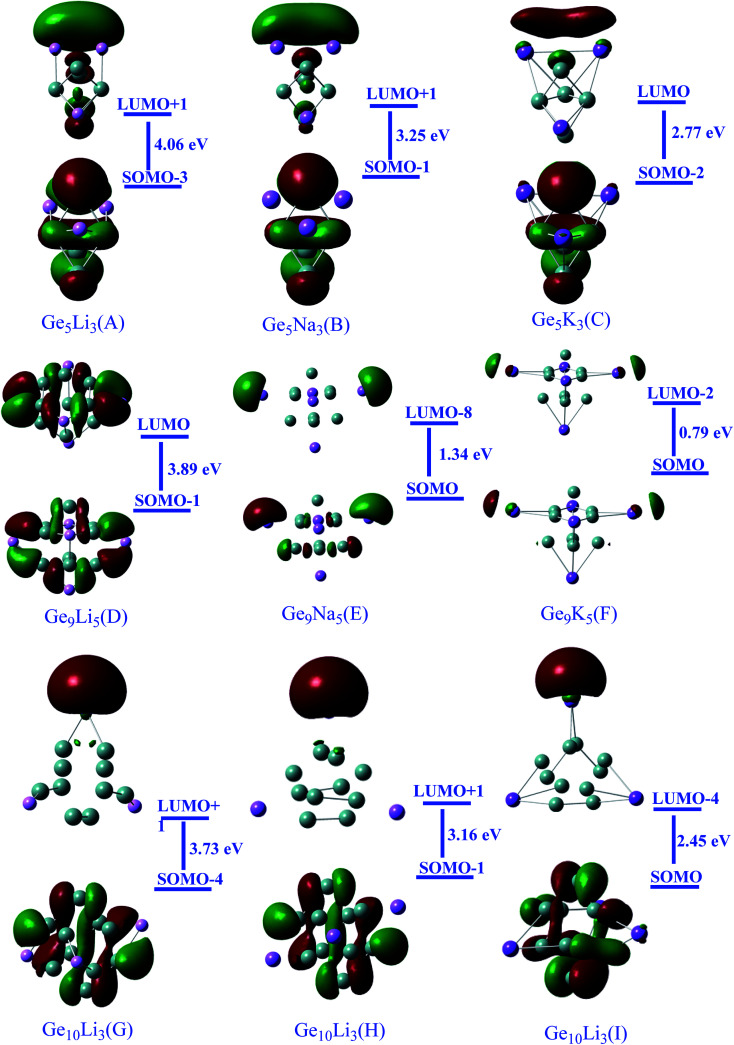
Representation of Frontier molecular orbital densities along with orbitals contribution of superalkali clusters (*iso*-value of 0.030).

Moreover, the excess electron character of clusters can be seen in the electronic density distribution (Fig. 2). The excess electron cloud is indicated by the electronic density of SOMO and LUMO scattered throughout the alkali metals that resemble to s-orbital. The LUMO electronic density for Ge_10_AM_3_ clusters wraps around the alkali metals, whereas the LUMO densities for Ge_9_AM_5_ clusters move away from the alkali metals.

### 3.4 TD-DFT analysis of clusters

**Fig. 3:**
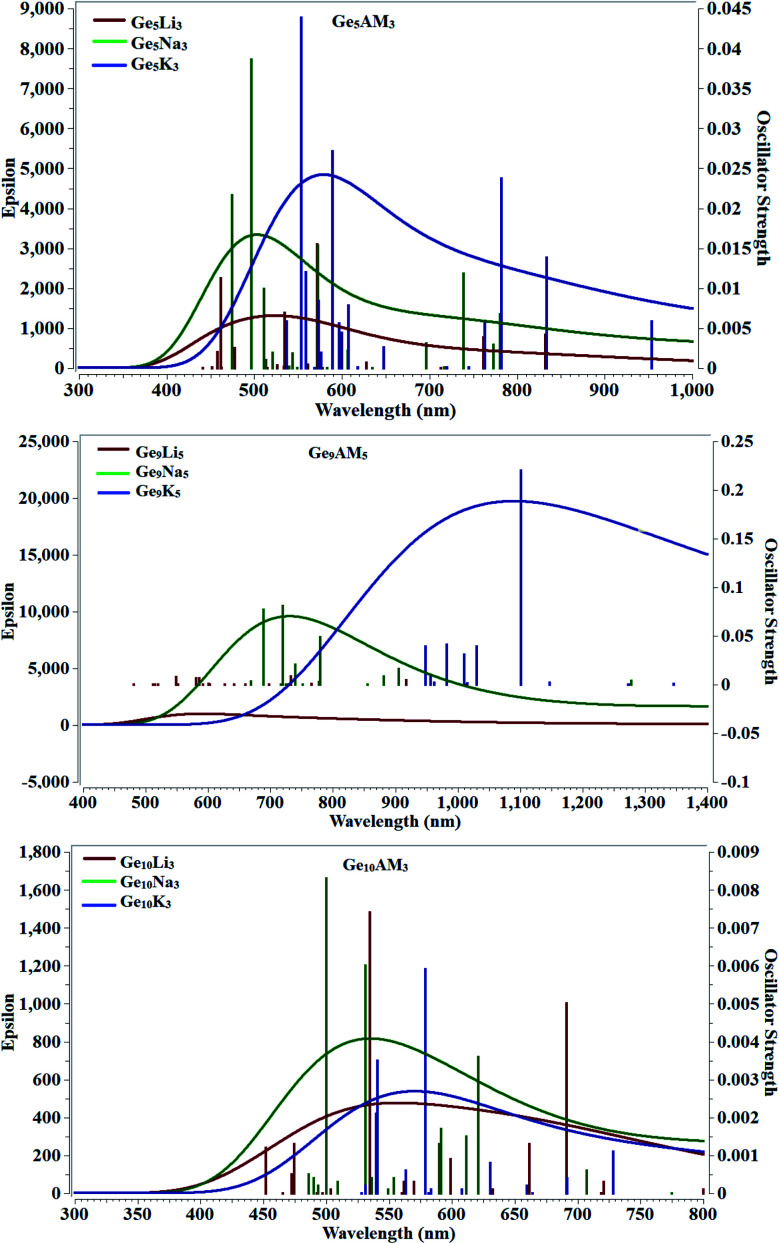
Absorbance spectra of Ge_5_AM_3_, Ge_9_AM_5_, and Ge_10_AM_3_.

Time-dependent density functional theory was used to examine the absorbance behavior of Ge_5_AM_3_, Ge_9_AM_5_, and Ge_10_AM_3_ superalkali clusters. For nonlinear optical applications, the cluster materials employed should be transparent in the used region. In the absorption study, we calculated the maximum absorbance, excitation energy, and oscillator strength (during the electronic transition). The computed excited state parameters for the studied superalkali clusters are given in Table 2. From the computed results one can observe that the studied clusters are completely transparent in the UV-region (<400 nm) and show broadband absorbance in the visible region. The longer absorbance wavelength for the Ge_5_AM_3_ (*λ*_mam_ = 572 nm) is observed for the Ge_5_Na_3_ superalkali cluster whereas the lowest absorbance maxima (*λ*_mam_ = 553 nm) is obtained for the Ge_5_K_3_ superalkali cluster (Fig. 3). However, the Ge_9_AM_5_ series shows higher absorbance maxima (*λ*_mam_ = 1101 nm) for Ge_9_K_5_ clusters. Similarly, for the second series, the highest absorbance maxima (*λ*_mam_ = 578 nm) is observed for the Ge_10_K_3_ cluster in the Ge_10_AM_3_ series. Overall, the clusters of Ge_9_AM_5_ series shows absorption maxima at a longer wavelength (bathochromic shift) whereas the Ge_5_AM_3_ and Ge_10_AM_3_ have slightly blue-shifted wavelengths. As a result of their total transparency under deep UV-region, the optoelectronic properties of the examined clusters fall under the UV-region. Furthermore, the excitation energies (Δ*E*) of Ge_5_AM_3_ (*i.e.* excitation of an electron from HOMO to LUMO) are also very small and ranges from 2.16 to 2.24 eV whereas the observed excitation energies (Δ*E*) for the Ge_9_AM_5_ are further reduced and lie in the range of 1.12 to 2.25 eV. Alternatively, the computed excitation energies of the Ge_10_AM_3_ series are slightly higher than those of Ge_9_AM_5_ and display a range of 2.14 to 2.47 eV. Moreover, the calculated oscillator strength (probability of absorbance during excitation) shows significant values for Ge_5_AM_3_ superalkali clusters while the Ge_9_AM_5_ and Ge_10_AM_3_ have slightly reduced values of oscillator strength (*f*_o_).

### 3.5 Dipole moment and change in dipole moment

Table 2 shows the computed values of mean dipole moment (*μ*_o_) and change in dipole moment (Δ*μ*). The magnitude of polarisation in clusters and the asymmetric charge distribution are shown by the significant values of dipole moments. For the Ge_5_AM_3_ series, the observed mean dipole moments range from 1.63 to 3.13 au. As the size of alkali metals grows larger (Li to K), the dipole moment gradually increase. Likewise, the calculated values of the dipole moment of Ge_9_AM_5_ range from 0.27 to 2.21 au. Thus the calculated values of dipole moment for the Ge_9_AM_5_ are slightly smaller than those of Ge_5_AM_3_ clusters. On the other hand, the computed mean dipole moments are enhanced for the Ge_10_AM_3_ series and the values lie in the range of 1.42 to 1.67 au. Hence, from the computed results, one can conclude that a significant dipole moment that is associated with the Ge_5_AM_3_ series would result in larger polarization. Our calculations also show that the studied alkali decorated zintl polyanions clusters possess polar bonds (asymmetric electronic density) that might be an important factor for imparting optical and nonlinear optical properties. Besides, the computed changes in dipole momen between the ground state and crucial excited state for Ge_5_AM_3_ clusters lies in the range of 1.05 to 2.34 au where the highest value of 2.34 au is observed for Ge_5_K_3_ while the lowest value of 1.05 au is obtained for the Ge_5_Li_3_ cluster. A similar decreasing trend of change in dipole moment is observed for the Ge_9_AM_5_ clusters. The value of excited-state dipole moment for the Ge_9_AM_5_ series lie in the range of 0.62 to 1.86 au. However, the computed values of the excited-state dipole moment are increasing with the increased size of alkali metals within clusters. Finally, the Ge_10_AM_3_ series of clusters show further decreased values of change in dipole moment. As a result of the fascinating electronic features of clusters examined, increased optical and nonlinear optical properties might be expected.

### 3.6 Static nonlinear optical properties

The alkali-like superatom clusters Ge_5_AM_3_, Ge_9_AM_5_, and Ge_10_AM_3_ examined here have an excess electron nature. As a result, large optical and nonlinear optical (NLO) responses are reasonable predictions. Literature reveals that compounds and clusters with excess electrons characteristics are significantly adopted for triggering nonlinear optical response.^19,36,39,56–65^ Hence, the calculated polarizability (*α*_o_), hyperpolarizability (*β*_o_), second hyperpolarizability (*γ*_o_), and associated electronic parameters are computed and given in Table 3. The calculated values of polarizability (linear optical response) of Ge_5_AM_3_ lie in the range of 3.70 × 10^−23^ to 5.3 × 10^−23^ esu. Similarly, the computed values of polarizability (*α*_o_) for Ge_9_AM_5_ series are significantly enhanced and lie in the range of 6.5 × 10^−23^ to 1.9 × 10^−21^ esu. Alternatively, the obtained values of polarizability of Ge_10_AM_3_ series are slightly smaller than those of Ge_9_AM_5_ and lie in range of 60 × 10^−24^ to 71 × 10^−24^ au. The increasing trend of polarizability may be seen as the size of alkali metals increases (Li to K). Overall, the highest value of 1.9 × 10^−21^ esu is obtained for *F* wheres the lowest value of 37 × 10^−24^ esu is observed for A. Furthermore, the significant polarizability values obtained demonstrate the amount of polarity within the examined clusters. Asymmetric distribution of charges and electronic densities inside clusters also contributed to the higher linear optical response.

**Table 3:** Polarizability (*α*_o_ in ×10^−24^ esu), first static hyperpolarizability (*β*_o_ in ×10^−33^ esu) scattering hyperpolarizability (*β*_vec_ in ×10^−33^ esu), static second hyperpolarizability (*γ*_o_ in ×10^−40^ esu), HOMO–LUMO gaps (*E*_H–L_ in au), and vertical ionization potential (VIP in au) of Ge_5_AM_3_, Ge_9_AM_5_ and Ge_10_AM_3_ superalkali clusters

Superalkalis	*α*_o_	*β*_o_	*β*_vec_	*γ*_o_	*E*_H–L_	VIP
**Ge**_**5**_ **AM**_**3**_						
Ge_5_Li_3_ (A)	3.7 × 10^−23^	3.44 × 10^−29^	3.39 × 10^−30^	1.37 × 10^−34^	4.04	5.49
Ge_5_Na_3_ (B)	4.5 × 10^−23^	1.01 × 10^−27^	9.94 × 10^−30^	5.5 × 10^−34^	3.25	4.69
Ge_5_K_3_ (C)	5.3 × 10^−23^	3.41 × 10^−28^	2.97 × 10^−29^	2.80 × 10^−33^	2.77	3.91

**Ge**_**9**_ **AM**_**5**_						
Ge_9_Li_5_ (D)	6.5 × 10^−23^	1.80 × 10^−28^	1.81 × 10^−29^	4.07 × 10^−34^	3.89	4.36
Ge_9_Na_5_ (E)	3.6 × 10^−22^	1.57 × 10^−26^	1.57 × 10^−26^	2.15 × 10^−30^	1.34	2.81
Ge_9_K_5_ (F)	1.9 × 10^−21^	8.99 × 10^−26^	8.99 × 10^−27^	7.68 × 10^−34^	0.79	2.15

**Ge**_**10**_ **AM**_**3**_						
Ge_10_Li_3_ (G)	6.0 × 10^−23^	1.88 × 10^−29^	1.88 × 10^−29^	2.26 × 10^−34^	3.73	4.98
Ge_10_Na_3_ (H)	6.8 × 10^−23^	3.87 × 10^−29^	3.87 × 10^−29^	3.87 × 10^−34^	3.16	4.34
Ge_10_K_3_ (I)	7.1 × 10^−23^	4.57 × 10^−29^	4.57 × 10^−29^	7.68 × 10^−34^	2.45	3.50

Furthermore, the calculated values of static hyperpolarizability (*β*_o_) lie in the range of 3.44 × 10^−29^ to 8.99 × 10^−26^ esu. The calculated static first and second hyperpolarizabilities values are significant. Hyperpolarizability values in the proposed superalkali clusters follow the order Ge_9_AM_5_ > Ge_5_AM_3_ > Ge_10_AM_3._ The hyperpolarizability values obtained for the Ge_5_M_3_ series rise monotonically with alkali metal size. The highest value of 3.41 × 10^−28^ esu is observed for the C cluster while the lowest value (3.44 × 10^−29^ esu) is calculated for the A cluster. Similarly, the obtained values of the *β*_o_ for the Ge_9_AM_5_ series range from 1.80 × 10^−28^–8.99 × 10^−26^ esu. With the increased metal size and the number of alkali metals in the Ge_9_AM_5_ clusters, there is a huge increase in *β*_o_ values. In particular, the Ge_9_K_5_ shows a remarkable *β*_o_ value (8.99 × 10^−26^ esu) which may be attributed to the larger size of alkali metal (K). In comparison, the Ge_10_AM_3_ (AM = Li, Na, and K) clusters have range of 1.88 × 10^−29^ to 4.57 × 10^−29^, which is lower than the Ge_9_AM_5_ and Ge_5_AM_3_ clusters. In our designed and studied clusters the hyperpolarizability response is quite larger than previously reported Li_*n*_F (*n* = 2–5) superalkali clusters,^39^ and M_2_OCN & M_2_NCO (M = Li, Na, K) clusters.^64^ Excess electron nature might account for considerable hyperpolarizability response found in these clusters. Furthermore, the studied fabulous electronic properties contribute to the hyperpolarizabilities values. As a result, decreased *E*_H–L_ gaps and ionization potential (IP) with increasing alkali metals (AM) size ultimately prompt the *β*_o_ response.

We used a conventional two model with the sum-over-state (SOS) method to develop a full understanding of hyperpolarizability and its governing factors. The two-level model can be written as follows: *β*_tl_ = Δ*μ* × *f*_o_/Δ*E*^3^

**Fig. 4:**
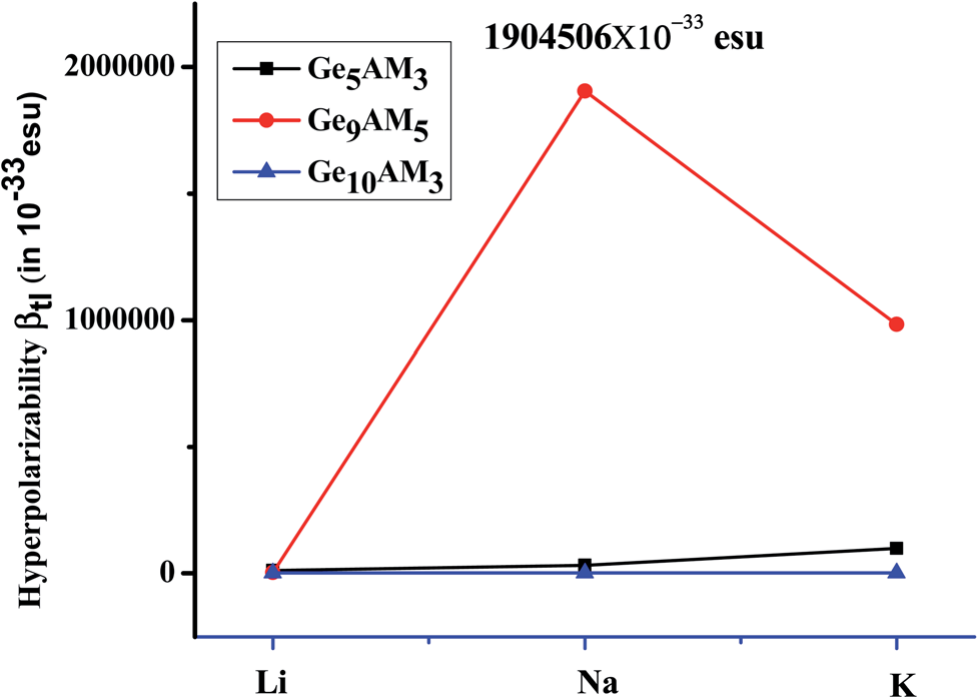
Representation of *β*_tl_ for Ge_5_AM_3_, Ge_9_AM_5,_ and Ge_10_AM_3_ superalkali clusters.

Where the Δ*μ*, *f*_o_, and Δ*E* are changes in dipole moment, oscillator strength, and excitation energy for crucial excitation (excitation with maximum oscillator strength). From the above model, one can observe that *β*_tl_ has a direct relation with change in dipole moment and oscillator strength (*f*_o_) while it is inversely related with cubic of excitation energy (Δ*E*). The obtained values of *β*_tl_ range from 1.03 × 10^−29^–1.90 × 10^−27^ esu (Table 4). The Ge_9_AM_5_ has the largest *β*_tl_ values in the examined clusters series, which is comparable to *β*_o_. For the first series (Ge_5_AM_3_), the *β*_tl_ is increased with an increase in Δ*μ* and oscillator strength. In this series, Ge_5_K_3_ shows a significant *β*_tl_ response which may be attributed to its noticeable change in dipole moment (2.34 au) and small excitation energy (1.58 eV). As a result, excitation energy (*E*) is considered to be extremely vital in impacting hyperpolarizability response. A similar trend is observed for the second series (Ge_9_AM_5_) where the Ge_9_Na_5_ shows a notable value (1.90 × 10^−27^ esu) of *β*_tl_ which is due to small excitation energy (transition energy) and higher change in dipole moment (2.49 au). Likewise, *β*_tl_ values for the Ge_10_AM_3_ series, there is an increase *β*_tl_ with reduced excitation energy (Δ*E*). Hence, the excitation energy is deciding factor in triggering the hyperpolarizability response from the two-level model where its effect is inversely related to *β*_tl_. Furthermore, the estimated *β*_o_ and *β*_tl_ values are highly correlated, providing additional insight into the hyperpolarizability response. From the plotted graph (Fig. 4), one can observe that overall, the *β*_tl_ values increase dramatically for the Ge_9_Na_5_ cluster.

**Table 4:** Computed hyperpolarizability from the two-level model (*β*_tl_ in ×10^−33^ esu), change in dipole moment (Δ*μ* in au), excitation energy (Δ*E* in eV), oscillator strength (*f*_o_ in au) hyper Rayleigh scattering (*β*_HRS_ in ×10^−33^ esu), and depolarization ratio (DR in au) of superalkali clusters

Superalkalis	*β*_tl_	Δ*μ*	Δ*E*	*f*_o_	*β*_HRS_	DR
**Ge**_**5**_ **AM**_**3**_						
Ge_5_Li_3_ (A)	1.03 × 10^−29^	1.05	2.16	0.05	1.31 × 10^−29^	6.87
Ge_5_Na_3_ (B)	3.11 × 10^−29^	1.14	1.67	0.04	4.23 × 10^−29^	5.61
Ge_5_K_3_ (C)	9.79 × 10^−29^	2.34	1.58	0.16	1.32 × 10^−28^	6.41

**Ge**_**9**_ **AM**_**5**_						
Ge_9_Li_5_ (D)	2.90 × 10^−30^	0.62	1.69	0.01	1.13 × 10^−29^	2.44
Ge_9_Na_5_ (E)	1.90 × 10^−27^	2.49	0.01	0.002	9.61 × 10^−27^	2.41
Ge_9_K_5_ (F)	9.83 × 10^−28^	1.86	0.75	0.06	5.03 × 10^−26^	2.45

**Ge**_**10**_ **AM**_**3**_						
Ge_10_Li_3_ (G)	1.54 × 10^−30^	0.62	1.87	0.01	1.21 × 10^−29^	2.37
Ge_10_Na_3_ (H)	1.79 × 10^−30^	0.65	2.02	0.21	2.08 × 10^−29^	2.45
Ge_10_K_3_ (I)	9.64 × 10^−31^	0.49	1.86	0.11	2.73 × 10^−29^	2.57

Projection of hyperpolarizability on dipole moment vector is also evaluated through *β*_vec_ simulations. The *β*_vec_ is a vector part of hyperpolarizability and is very important to characterize the nonlinearity of molecules and clusters. The calculated *β*_vec_ values are given in Table 3. From Table 3, the observed *β*_vec_ response of designed clusters strongly correlates with total hyperpolarizability which indicates the dipole moment has the same direction projection of hyperpolarizability (*β*_o_). Furthermore, the quite comparable values of first hyperpolarizabilities with *β*_vec_ also indicate that the charge transfer is parallel to the molecular dipole moments. The resemblance of *β*_o_ and *β*_vec_ also suggests that these clusters can be synthesized in laboratory.

We also carried out calculations for estimation of static second hyperpolarizability (*γ*_o_) which is a third rank tensor (*χ*^3^). The obtained values of *γ*_o_ for the Ge_5_AM_3_ range from 1.37 × 10^−34^–2.80 × 10^−33^ esu which shows an increasing trend with the increased size of alkali metals. Similarly, for the second series (Ge_9_AM_5_), the *γ*_o_ response increases up to 2.15 × 10^−30^ esu for E cluster. The *γ*_o_ values for the Ge_10_AM_3_ superalkali clusters, on the other hand, decrease considerably. Clusters with a higher number of alkali metals (AM) have a substantial *γ*_o_ response. Likewise, the *γ*_o_ response for the Ge_10_AM_3_ clusters lie in the range of 2.26 × 10-^34^ to 7.68 × 10^−34^ esu and a slight increase is observed with growing alkali metals size. Hence, the *γ*_o_ values vary in order of Ge_9_AM_5_ > Ge_5_AM_3_ > Ge_10_AM_3._

### 3.7 Hyper Rayleigh scattering measurement (*β*_HRS_)

The hyperpolarizability of nonlinear optical molecules can be determined using hyper-Rayleigh scattering. The *β*_HRS_ is a widely used method for determining the nonlinear optical characteristics of centrosymmetric molecules with zero dipole moment. At the same level of theory, we theoretically evaluated the *β*_HRS_ response for these clusters. Overall, the calculated highest value (5.03 × 10^−26^ esu) of *β*_HRS_ is obtained for Ge_9_K_5_ while the lowest value 1.13 × 10^−29^ esu is observed for D. For the Ge_5_AM_3_ series *β*_HRS_ lie in the range of 1.31 × 10^−29^ esu to 1.32 × 10^−28^ esu, and are increased with size (Li to K). In the Ge_9_AM_5_ series, the usual trend of beta-HRS value of Ge_9_Li_5_ may be attributed to the small size of Li-atom. Similarly, other nonlinear optical parameters for Ge_9_Li_5_ are also small. It has been reported in the literature previously that dominating factor changes from structure to structure (and it ultimately leads to irregular trends). However, factors affecting the *β*_HRS_ response might be the same as total hyperpolarizability values. A similar increasing trend of *β*_HRS_ with the size of metal (AM) can be seen for the second series of clusters(Ge_9_AM_5_). Furthermore, *β*_HRS_ values do not increase with clusters size, rather these show dependence upon the size of and the number of alkali metals. As a result, the *β*_HRS_ values for the Ge_10_AM_3_ become slightly smaller, ranging from 1.22 × 10^−29^ to 2.73 × 10^−29^ esu. Interestingly, the Ge_9_K_5_ exhibits a significant HRS response which suggests better NLO properties. Additionally, the depolarization ratio (DR) is higher for Ge_5_AM_3_ clusters and increases up to 6.87 au for Ge_5_Li_3_.

#### 3.7.1 Dynamic nonlinear optical properties

The frequency-dependent NLO properties are the fundamental molecular parameters that are required for the description of many nonlinear optical phenomena. The theoretical understanding and accurate determination of the frequency-dependent hyperpolarizabilities *β*(*ω*) and second hyperpolarizabilities *γ*(*ω*) are therefore crucial to classify nonlinearity of materials. The frequency-dependent NLO response is estimated at dispersion frequencies of 532 and 1064 nm, and we calculated electro-optic pockel's effect (EOPE) with *β*(−*ω*;*ω*,0) and electric field induced second harmonic generation (EFSHG) with *β*(−2*ω*;*ω*,*ω*) at applied frequency. The obtained values of electro-optical pockel's effect (EOPE) for Ge_5_AM_3_ clusters at dispersion frequency of 532 nm range from. The EOPE effect is much pronounced at a small dispersion frequency of 532 nm. Similarly, the obtained dynamic hyperpolarizabilities *β*(−2*ω*;*ω*,*ω*) values noticeably at smaller frequencies. Overall, the highest values (3.54 × 10^−26^ esu) of EOPE is obtained for C whereas the lowest value of 3.02 × 10^−28^ esu is observed for D (Table 5). The is a gradual increase in *β*(*ω*) with an increased size of alkali metals metal. Both EOPE and SHG show significant values at smaller applied frequencies, and their values slightly decrease at higher dispersion frequency (1064 nm).

**Table 5:** Frequency-dependent hyperpolarizability *β*(*ω*) in form of electro-optic pockel's effect (EOPE) *β*(−*ω*;*ω*,0) in ×10^−33^ esu, and electric field induced second harmonic generation (EFSHG) with *β*(2 − *ω*;*ω*,*ω*) in ×10^−33^ esu at *ω* = 532 nm and *ω* = 1064 nm

Superalkalis	*ω* = 0.0856 au (532 nm)		*ω* = 0.0428 au (1064 nm)	
	*β*(*−ω*;*ω*,0)	*β*(*−*2*ω*;*ω*,0)	*β*(*−ω*;*ω*,0)	*β*(*−*2*ω*;*ω*,0)
**Ge**_**5**_ **AM**_**3**_				
Ge_5_Li_3_ (A)	3.28 × 10^−27^	4.42 × 10^−27^	6.99 × 10^−29^	1.81 × 10^−28^
Ge_5_Na_3_ (B)	1.29 × 10^−27^	3.63 × 10^−27^	2.50 × 10^−28^	3.72 × 10^−28^
Ge_5_K_3_ (C)	3.54 × 10^−26^	1.30 × 10^−26^	2.24 × 10^−28^	9.50 × 10^−28^

**Ge**_**9**_ **AM**_**5**_				
Ge_9_Li_5_ (D)	3.02 × 10^−29^	3.28 × 10^−29^	7.86 × 10^−29^	1.47 × 10^−30^
Ge_9_Na_5_ (E)	7.68 × 10^−28^	4.42 × 10^−27^	1.56 × 10^−26^	2.94 × 10^−27^
Ge_9_K_5_ (F)	6.39 × 10^−27^	1.04 × 10^−26^	4.93 × 10^−29^	1.39 × 10^−27^

**Ge**_**10**_ **AM**_**3**_				
Ge_10_Li_3_ (G)	9.67 × 10^−29^	1.98 × 10^−28^	2.24 × 10^−29^	6.99 × 10^−29^
Ge_10_Na_3_ (H)	5.71 × 10^−28^	3.71 × 10^−28^	2.85 × 10^−29^	5.62 × 10^−29^
Ge_10_K_3_ (I)	1.21 × 10^−27^	1.04 × 10^−27^	5.87 × 10^−29^	2.42 × 10^−28^

The calculated frequency-dependent second hyperpolarizability *γ*(*ω*) that includes dc-Kerr *γ*^dc−Kerr^ (*ω*) = *γ*(−*ω*;*ω*,0,0) and second harmonic generation with *γ*^ESHG^(*ω*) = *γ*(−2*ω*;*ω*,*ω*,0) are calculated at the same level of theory. The calculated values of dynamic second hyperpolarizability are given in Table 6. The calculated dc-Kerr constant and EFSHG are higher at 532 nm and their values slightly decreased at higher dispersion frequency (1064 nm). Likewise, Ge_9_AM_5_ and Ge_10_AM_3_ are also larger at a small dispersion frequency of 532 nm. Hence, the studied clusters offer tremendous dynamic NLO properties at the smaller external applied frequency.

**Table 6:** Frequency-dependent second hyperpolarizability with dc-Kerr effect *γ* (−*ω*;*ω*,0,0) and electric field induced second harmonic generation (ESHG) *γ*(−2−*ω*;*ω*,*ω*,0) in ×10^−40^ esu at *ω* = 532 and 1064 nm

Superalkalis	*ω* = 0.0856 au (532 nm)		*ω* = 0.0428 au (1064 nm)	
	*γ*(−*ω*;*ω*,0,0)	*γ*(−2*ω*;*ω*,*ω*,0)	*γ*(−*ω*;*ω*,0,0)	*γ*(−2*ω*;*ω*,*ω*,0)
**Ge**_**5**_ **AM**_**3**_				
Ge_5_Li_3_ (A)	7.84 × 10^−31^	1.37 × 10^−30^	3.39 × 10^−34^	1.40 × 10^−30^
Ge_5_Na_3_ (B)	1.30 × 10^−32^	1.64 × 10^−31^	2.45 × 10^−33^	2.31 × 10^−33^
Ge_5_K_3_ (C)	5.43 × 10^−36^	2.52 × 10^−29^	7.37 × 10^−32^	2.09 × 10^−31^

**Ge**_**9**_ **AM**_**5**_				
Ge_9_Li_5_ (D)	1.36 × 10^−33^	5.74 × 10^−32^	3.16 × 10^−32^	1.19 × 10^−33^
Ge_9_Na_5_ (E)	1.76 × 10^−29^	8.80 × 10^−30^	1.58 × 10^−31^	1.03 × 10^−32^
Ge_9_K_5_ (F)	5.23 × 10^−32^	2.53 × 10^−32^	2.15 × 10^−30^	3.76 747 508 × 10^−32^

**Ge**_**10**_ **AM**_**3**_				
Ge_10_Li_3_ (G)	8.45 × 10^−32^	1.24 × 10^−31^	3.24 × 10^−40^	5.28 × 10^−34^
Ge_10_Na_3_ (H)	1.22 × 10^−32^	6.84 × 10^−34^	1.72 × 10^−32^	2.41 × 10^−33^
Ge_10_K_3_ (I)	5.23 × 10^−32^	2.53 × 10^−32^	2.15 × 10^−30^	3.76 × 10^−32^

## 4 Conclusion

In the summary, we explored the zintl-based superalkali for geometric, electronic and nonlinear optical properties. The studied zintl superalkali clusters Ge_5_AM_3_, Ge_9_AM_5_, and Ge_10_AM_3_ (AM = Li, Na, K) belong to excess electron compounds. These are superalkali clusters as their calculated vertical ionization potential (VIP) values are smaller than Li atom (5.39 eV). The calculated significant VIP values suggest their electronic stability. The calculated chemical hardness (*η*) lie in the range of 2.13 to 4.51 eV, and Ge_9_AM_5_ shows the higher softness among the series. There is a significant charge (positive in magnitude) on alkali metals, and the charge is transferred from alkali to Ge-atom. The charge is transferred from alkali metals to Ge-atoms within the clusters. There is a notable reduction in *E*_H–L_ (0.79–4.04 eV) which reveals their conductive applications. These clusters are completely transparent in the deep UV region, and show absorption maxima (*λ*_max_) at the longer wavelength. Being excess electron compounds these clusters shows remarkable hyperpolarizability response up to 8.99 × 10^−26^ esu where the static second hyperpolarizability (*γ*_o_) value recorded up to 2.15 × 10^−30^ esu for Ge_9_AM_5_ clusters. The adopted two-level model study reveals the controlling factors of hyperpolarizability. The obtained significant *β*_tl_ value of 1.90 × 10^−27^ esu may attributed to smaller excitation energy (0.01 eV) The frequency-dependent hyperpolarizabilities and second hyperpolarizabilities values are much higher at smaller dispersion frequencies (*ω* = 532 nm). Moreover, the hyper Rayleigh scattering (*β*_HRS_) increases up to 5.03 × 10^−27^ esu for the Ge_9_K_5_ cluster.

## Conflicts of interest

There are no conflicts to declare.

## Supplementary Material

RA-012-D1RA08192F-s001
