# Supplementary material for: Germanium-based superatom clusters as excess electron compounds with significant static and dynamic NLO response; a DFT study
Source: RSC Adv. 2021 Dec 21;12(1):365–77. doi: 10.1039/d1ra08192f (PMC8978613; doi:10.1039/d1ra08192f)
Supplement: RA-012-D1RA08192F-s001 [file RA-012-D1RA08192F-s001.pdf]

Electronic Supplementary Material (ESI) for RSC Advances.
This journal is © The Royal Society of Chemistry 2021

# Electronic Supplementary information (ESI)

## Germanium-based superatom clusters as excess electron compounds with significant static and dynamic NLO response; A DFT Study

Atazaz Ahsin, Ahmed Bilal Shah, Khurshid Ayub*

Department of Chemistry, COMSATS University Islamabad, Abbottabad Campus, Abbottabad, KPK, 22060, Pakistan

Correspondence:

khurshid@cuiatd.edu.pk (K. A.)

## 1. Optimized geometric analysis

The obtained geometric parameters of these clusters are given in Table 1 (ESI). The obtained point group symmetry of $Ge_5AM_3$, $Ge_9AM_5$ and $Ge_{10}AM_3$ (where AM = Li, Na, K) are $C_S$, $C_{4V}$, and $C_2$ respectively. The calculated equilibrium bond length ($X_{Ge\text{-}Ge}$) in the $Ge_5AM_3$ series are lie in the range of 2.53 to 2.54 Å. Similarly, the obtained values of $X_{Ge\text{-}Ge}$ bond length in the $Ge_9AM_5$ series are slightly enhanced and lie in the range of 2.57 to 2.68 Å. However, the calculated average values of $X_{Ge\text{-}Ge}$ bond lengths between germanium atoms in $Ge_{10}AM_3$ clusters are further increased. Thus the observed trend average computed bond length (Ge-Ge) within the clusters series is $Ge_5AM_3 < Ge_9AM_5 < Ge_{10}AM_3$. One can also conclude that, with the increased cluster size, there is a gradual enhancement in bond distance between the germanium atom from $Ge_5$ to $Ge_9$ and then to $Ge_{10}$.

Furthermore, the computed average bond lengths ($X_{Ge\text{-}AM}$) for $Ge_5AM_3$ clusters lie in the range of 2.51 to 3.34 Å. In the series ($Ge_5AM_3$), the observed bond length increases monotonically with increased metal sized. Thus highest computed bond length $X_{Ge\text{-}AM}$ (where AM = Li, Na, K) value of 3.34 Å is observed for the $Ge_5K_3$ cluster while the lowest of 2.51 Å is indicated for the $Ge_5Li_3$ cluster. Similarly, the calculated value of ($X_{Ge\text{-}AM}$) bond lengths for the of zintl $Ge_9AM_5$ lie in the rage of 2.57 to 3.33 Å. The highest obtained value of 3.33 Å for $Ge_9K_5$ is attributed to large sized alkali metal.

**Table S1:** Point group symmetry, average computed $X_{Ge\text{-}Ge}$ bond length (in Å), average computed $X_{Ge\text{-}AM}$ bond lengths (in Å where AM=Li, Na, K), binding energy per atom ($E_B$ in kcal mol$^{-1}$), and total energy ($E_{tot}$ in hartree) of studied superalkali clusters.

| **$Ge_5AM_3$** | | | | |
|---|---|---|---|---|
| **Superalkalis** | **Symmetry** | **$X_{Ge\text{-}Ge}$** | **$X_{Ge\text{-}AM}$** | **$E_{tot}$** |
| $Ge_5Li_3$ (A) | $C_S$ | 2.56 | 2.51 | 10408.09 |
| $Ge_5Na_3$ (B) | $C_S$ | 2.53 | 2.84 | 10872.40 |
| $Ge_5K_3$ (C) | $C_S$ | 2.52 | 3.34 | 12185.41 |
| **$Ge_9AM_5$** | | | | |
| $Ge_9Li_5$ (D) | $C_{4V}$ | 2.57 | 2.57 | 18731.62 |
| $Ge_9Na_5$ (E) | $C_{4V}$ | 2.66 | 3.01 | 19505.47 |
| $Ge_9K_5$ (F) | $C_{4V}$ | 2.68 | 3.33 | 21693.84 |

| $Ge_{10}AM_3$ | | | | |
|---|---|---|---|---|
| $Ge_{10}Li_3$ (G) | $C_2$ | 2.84 | 2.56 | 20793.65 |
| $Ge_{10}Na_3$ (H) | $C_2$ | 2.65 | 2.94 | 21257.97 |
| $Ge_{10}K_3$ (I) | $C_2$ | 2.73 | 3.24 | 22571.01 |

2. **Optimized geometric coordinates at CAM-B3LYP/6-311+G(d,p)**
   The optimized geometries and their cartesian coordinates at above functional are given in Table 2.

**Table S2:** Optimized geometric coordinates of $Ge_5AM_3$, $Ge_9AM_5$, $Ge_{10}AM_3$ clusters

**$Ge_5AM_3$ (where AM=Li, Na K)**

**A**

| | | | | | |
|---|---|---|---|---|---|
| 1 | 32 | 0 | -1.884226 | 0.850537 | 0.000000 |
| 2 | 32 | 0 | -0.389141 | -0.677590 | 1.349466 |
| 3 | 32 | 0 | -0.389141 | -0.677590 | -1.349466 |
| 4 | 32 | 0 | 0.840445 | 1.340237 | 0.000000 |
| 5 | 32 | 0 | 1.891553 | -0.894711 | 0.000000 |
| 6 | 3 | 0 | 0.037077 | -2.981132 | 0.000000 |
| 7 | 3 | 0 | -0.389141 | 1.805857 | 2.325246 |
| 8 | 3 | 0 | -0.389141 | 1.805857 | -2.325246 |

---------------------------------------------------------------------

**B**

| | | | | | |
|---|---|---|---|---|---|
| 1 | 32 | 0 | -2.124191 | 0.843194 | 0.000000 |
| 2 | 32 | 0 | -0.274994 | -0.225238 | 1.380866 |
| 3 | 32 | 0 | -0.274994 | -0.225238 | -1.380866 |
| 4 | 32 | 0 | 1.554371 | 0.977147 | 0.000000 |
| 5 | 32 | 0 | 1.317566 | -1.618584 | 0.000000 |
| 6 | 11 | 0 | -0.274994 | 2.394316 | 1.429215 |
| 7 | 11 | 0 | -1.559439 | -1.904018 | 0.000000 |
| 8 | 11 | 0 | -0.274994 | 2.394316 | -1.429215 |

---------------------------------------------------------------------

**C**

| | | | | | |
|---|---|---|---|---|---|
| 1 | 32 | 0 | -2.053163 | 0.690772 | 0.000000 |
| 2 | 32 | 0 | -0.176512 | -0.343358 | 1.363159 |
| 3 | 32 | 0 | -0.176512 | -0.343358 | -1.363159 |
| 4 | 32 | 0 | 1.676226 | 0.862787 | 0.000000 |
| 5 | 32 | 0 | 1.428969 | -1.735165 | 0.000000 |
| 6 | 19 | 0 | -1.680455 | -2.387954 | 0.000000 |
| 7 | 19 | 0 | -0.176512 | 2.456993 | 1.732894 |

| | | | | | |
|---|---|---|---|---|---|
| 8 | 19 | 0 | -0.176512 | 2.456993 | -1.732894 |

---------------------------------------------------------------------

$Ge_9AM_5$

D

| | | | | | |
|---|---|---|---|---|---|
| 1 | 32 | 0 | 1.877671 | 0.000000 | -1.399972 |
| 2 | 32 | 0 | -1.410469 | 1.410469 | 0.807043 |
| 3 | 32 | 0 | -1.877671 | 0.000000 | -1.399972 |
| 4 | 32 | 0 | 0.000000 | -1.877671 | -1.399972 |
| 5 | 32 | 0 | 0.000000 | 1.877671 | -1.399972 |
| 6 | 32 | 0 | 0.000000 | 0.000000 | 2.533846 |
| 7 | 32 | 0 | 1.410469 | -1.410469 | 0.807043 |
| 8 | 32 | 0 | -1.410469 | -1.410469 | 0.807043 |
| 9 | 32 | 0 | 1.410469 | 1.410469 | 0.807043 |
| 10 | 3 | 0 | 0.000000 | 3.762213 | 0.394238 |
| 11 | 3 | 0 | 0.000000 | 0.000000 | -3.306325 |
| 12 | 3 | 0 | 3.762213 | 0.000000 | 0.394238 |
| 13 | 3 | 0 | -3.762213 | 0.000000 | 0.394238 |
| 14 | 3 | 0 | 0.000000 | -3.762213 | 0.394238 |

---------------------------------------------------------------------

E

| | | | | | |
|---|---|---|---|---|---|
| 1 | 32 | 0 | -0.000044 | -1.296794 | 1.891368 |
| 2 | 32 | 0 | 1.594194 | 0.819133 | -1.285309 |
| 3 | 32 | 0 | -0.000037 | -1.299661 | -1.889546 |
| 4 | 32 | 0 | -1.801465 | -1.497280 | 0.000983 |
| 5 | 32 | 0 | 1.801365 | -1.497385 | 0.000990 |
| 6 | 32 | 0 | 0.000070 | 2.439021 | -0.001499 |
| 7 | 32 | 0 | -1.594464 | 0.821109 | 1.284116 |
| 8 | 32 | 0 | -1.594127 | 0.819221 | -1.285318 |
| 9 | 32 | 0 | 1.594512 | 0.821022 | 1.284119 |
| 10 | 11 | 0 | 3.874235 | 0.104991 | 0.000071 |
| 11 | 11 | 0 | 0.000040 | 0.873018 | -3.285682 |
| 12 | 11 | 0 | 0.000022 | 0.877962 | 3.284110 |
| 13 | 11 | 0 | -3.874220 | 0.105245 | 0.000031 |
| 14 | 11 | 0 | -0.000097 | -3.204240 | 0.002418 |

---------------------------------------------------------------------

F

| | | | | | |
|---|---|---|---|---|---|
| 1 | 32 | 0 | -0.000023 | 1.797508 | 1.341929 |
| 2 | 32 | 0 | 1.428077 | -1.390586 | -0.836325 |
| 3 | 32 | 0 | -0.000090 | -1.798469 | 1.338650 |
| 4 | 32 | 0 | -1.904868 | -0.001538 | 1.363939 |
| 5 | 32 | 0 | 1.904707 | -0.001564 | 1.364082 |
| 6 | 32 | 0 | 0.000090 | 0.002559 | -2.523112 |
| 7 | 32 | 0 | -1.427761 | 1.391643 | -0.832651 |
| 8 | 32 | 0 | -1.428051 | -1.390481 | -0.836404 |

| | | | | | |
|---|---|---|---|---|---|
| 9 | 32 | 0 | 1.427892 | 1.391549 | -0.832578 |
| 10 | 19 | 0 | 4.466674 | 0.001246 | -0.919075 |
| 11 | 19 | 0 | 0.000220 | 4.436661 | -0.733006 |
| 12 | 19 | 0 | -4.466471 | 0.001591 | -0.919423 |
| 13 | 19 | 0 | -0.000152 | -4.437295 | -0.735491 |
| 14 | 19 | 0 | -0.000225 | -0.003250 | 4.069048 |

---------------------------------------------------------------------

**$Ge_0AM_3$**

**G**

| | | | | | |
|---|---|---|---|---|---|
| 1 | 32 | 0 | 2.142890 | 0.133790 | -0.775192 |
| 2 | 32 | 0 | -0.881017 | -1.887825 | 0.770452 |
| 3 | 32 | 0 | -2.142890 | -0.133790 | -0.775192 |
| 4 | 32 | 0 | 0.000000 | 1.288547 | -1.861020 |
| 5 | 32 | 0 | 0.000000 | -1.288547 | -1.861020 |
| 6 | 32 | 0 | -1.256068 | 0.453309 | 1.817801 |
| 7 | 32 | 0 | 0.881017 | 1.887825 | 0.770452 |
| 8 | 32 | 0 | -1.613176 | 2.366289 | -0.009281 |
| 9 | 32 | 0 | 1.256068 | -0.453309 | 1.817801 |
| 10 | 32 | 0 | 1.613176 | -2.366289 | -0.009281 |
| 11 | 3 | 0 | 0.000000 | 0.000000 | 4.104612 |
| 12 | 3 | 0 | -2.267041 | -2.716739 | -1.441740 |
| 13 | 3 | 0 | 2.267041 | 2.716739 | -1.441740 |

**H**

| | | | | | |
|---|---|---|---|---|---|
| 1 | 32 | 0 | 2.216692 | 0.563433 | -0.580820 |
| 2 | 32 | 0 | -0.430143 | -1.998719 | 0.767084 |
| 3 | 32 | 0 | -2.216692 | -0.563433 | -0.580820 |
| 4 | 32 | 0 | 0.000000 | 1.261030 | -1.856468 |
| 5 | 32 | 0 | 0.000000 | -1.261030 | -1.856468 |
| 6 | 32 | 0 | -1.312450 | 0.272097 | 1.758832 |
| 7 | 32 | 0 | 0.430143 | 1.998719 | 0.767084 |
| 8 | 32 | 0 | -1.980202 | 1.965648 | -0.333863 |
| 9 | 32 | 0 | 1.312450 | -0.272097 | 1.758832 |
| 10 | 32 | 0 | 1.980202 | -1.965648 | -0.333863 |
| 11 | 11 | 0 | -1.858055 | -3.072946 | -1.242730 |
| 12 | 11 | 0 | 0.000000 | 0.000000 | 3.912281 |
| 13 | 11 | 0 | 1.858055 | 3.072946 | -1.242730 |

---------------------------------------------------------------------

**I**

| | | | | | |
|---|---|---|---|---|---|
| 1 | 32 | 0 | 2.142890 | 0.133790 | -0.775192 |
| 2 | 32 | 0 | -0.881017 | -1.887825 | 0.770452 |
| 3 | 32 | 0 | -2.142890 | -0.133790 | -0.775192 |
| 4 | 32 | 0 | 0.000000 | 1.288547 | -1.861020 |
| 5 | 32 | 0 | 0.000000 | -1.288547 | -1.861020 |
| 6 | 32 | 0 | -1.256068 | 0.453309 | 1.817801 |

| | | | | | |
|---|---|---|---|---|---|
| **7** | **32** | **0** | **0.881017** | **1.887825** | **0.770452** |
| **8** | **32** | **0** | **-1.613176** | **2.366289** | **-0.009281** |
| **9** | **32** | **0** | **1.256068** | **-0.453309** | **1.817801** |
| **10** | **32** | **0** | **1.613176** | **-2.366289** | **-0.009281** |
| **11** | **19** | **0** | **-2.267041** | **-2.716739** | **-1.441740** |
| **12** | **19** | **0** | **2.267041** | **2.716739** | **-1.441740** |
| **13** | **19** | **0** | **0.000000** | **0.000000** | **4.104612** |

---------------------------------------------------------------------
